# Structural basis of the residence time of adenosine A_2A_ receptor ligands revealed by NMR

**DOI:** 10.1039/d5sc02398j

**Published:** 2025-08-29

**Authors:** Takumi Ueda, Tomoki Tsuchida, Masatoshi Kurita, Takuya Mizumura, Shunsuke Imai, Yutaro Shiraishi, Yutaka Kofuku, Shuhei Miyakawa, Kaori Fukuzawa, Koh Takeuchi, Ichio Shimada

**Affiliations:** a Graduate School of Pharmaceutical Sciences, The University of Tokyo 7-3-1 Hongo Bunkyo Tokyo 113-0033 Japan; b Graduate School of Pharmaceutical Sciences, The University of Osaka 1-6, Yamadaoka Suita Osaka Japan; c RIKEN Center for Integrative Medical Sciences 1-7-22, Suehiro-cho, Tsurumi-ku Yokohama Kanagawa 230-0045 Japan ichio.shimada@riken.jp; d Graduate School of Integrated Sciences for Life, Hiroshima University 1-4-4 Kagamiyama Higashi-Hiroshima City Hiroshima 739-8528 Japan

## Abstract

Residence time, which refers to the average duration a drug remains bound to its receptor, is a crucial parameter in determining its pharmacological effects. However, the mechanisms governing the residence time of G protein-coupled receptor (GPCR) ligands remain unclear. In this study, we observed NMR signals from the methyl groups of alanine and methionine located at the intersection of the binding cavity and extracellular loops of A_2A_AR under conditions where E165Q and T256A mutations led to reduced residence times. Our NMR analysis revealed that the spatial arrangement surrounding the E165–H264 salt bridge correlates with residence time. These findings provide quantitative insights into residence time and could assist in the development of drugs with optimized effects.

## Abstract 2

Our NMR analysis of adenosine A_2A_ receptor (A_2A_AR) revealed that spatial arrangement surrounding the E165–H264 salt bridge correlates with the residence time—the average time a drug remains bound to its receptor.

## Graphical abstract

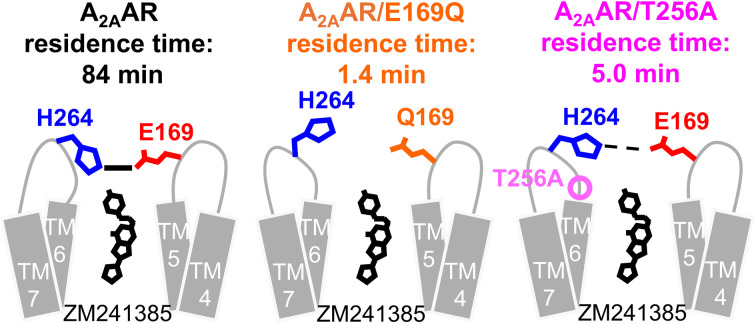

## Introduction

In the early stage of drug discovery campaigns, compounds are usually evaluated based on their equilibrium dissociation constant or its proxies, such as EC_50_ and IC_50_. However, drug concentrations in the human body fluctuate over time due to factors such as gastrointestinal absorption, tissue distribution, drug metabolism, and elimination processes, which may prevent the attainment or maintenance of binding equilibrium.^1^ Additionally, ligand-receptor binding kinetics can induce time-dependent biases in intracellular signaling patterns.^2,3^ Increasing evidence suggests that residence time, the average time a drug remains bound to its receptor, often determines the duration of target occupancy *in vivo* and correlates with the pharmacological actions of the drug.^1,4,5^ When achieving target selectivity is important, a drug with a longer residence time on one receptor can kinetically favour that receptor over others.^6^ Conversely, drugs with faster dissociation rates can increase the therapeutic index, defined as the ratio of a drug's toxic dose to its efficacious dose, when extended target receptor occupancy leads to toxicity.^6^

Adenosine A_2A_ receptor (A_2A_AR) is a class A GPCR that regulates inflammation, neurotransmission, blood flow, and immune responses.^7^ A_2A_AR ligands are used in the treatment of Parkinson's disease^8^ and myocardial perfusion imaging,^9^ and clinical evaluation of A_2A_AR ligands for cancer immunotherapy is ongoing.^10^ The residence time of A_2A_AR ligands determines the duration of sustained agonist responses,^11^ and plays a crucial role in their pharmacological effects.^12^ Therefore, understanding the relationship between residence time and the conformation of A_2A_AR would provide valuable insights into the mechanisms underlying residence time and aid in the development of drugs with optimal effects.

The three-dimensional structures of A_2A_AR bound to various ligands have been determined using X-ray crystallography and cryo-electron microscopy.^13–17^ In solution, A_2A_AR, like other GPCRs, exists in an equilibrium between multiple inactive and active conformations, with the populations and exchange rates determining signaling activities, as demonstrated by NMR and other spectroscopic studies.^18–33^

**Fig. 1:**
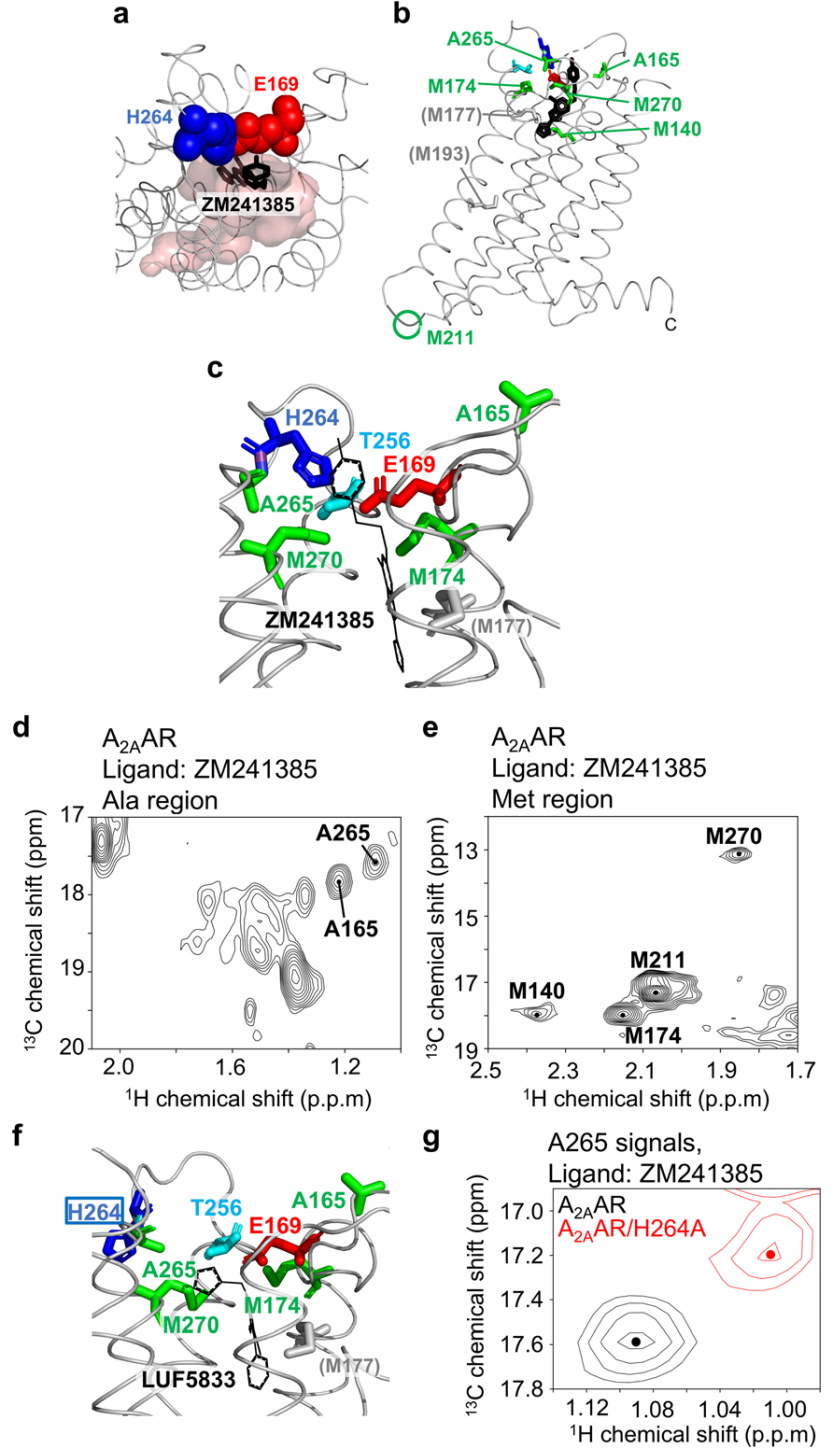
NMR signals of alanine and methionine methyl groups of A_2A_AR. (a) Closing of the ligand binding pocket by the E169–H264 salt bridge in the crystal structure of A_2A_AR (PDB code: 3EML). E169 and H264 are depicted by blue and red spheres, respectively, while the ligand-binding pocket is shown in pink. ZM241385 is depicted as black sticks. (b) Distribution of the observed residues in the crystal structure of A_2A_AR. The crystal structure of A_2A_AR with ZM241385 (PDB code: 3EML), is shown in ribbons in a side view, with the extracellular side at the top. Methionine residues, A165, and A265 are shown as green sticks. The region corresponding to M211, which is not observed in the crystal structure, is indicated by a green circle. M177 and M193, which were likely not observed due to line broadening of the resonances from these residues, are shown in grey. E169, T256, and H264 are depicted by blue, cyan, and red sticks, respectively. ZM241385 is represented by black sticks. (c) Magnified view of the region surrounding the E169–H264 salt bridge in A_2A_AR. The display style and coloring of A_2A_AR are identical to those in panel B. ZM241385 is represented by black lines. (d) and (e). ^1^H–^13^C HMQC spectra of [^2^H-8AA, αβγ-^2^H, methyl-^13^C-Met, α-^2^H, methyl-^13^C-Ala] A_2A_AR bound to ZM241385. Only the alanine and methionine methyl regions are shown in (d) and (e), respectively. The resonances from A165, A265, M140, M174, M211, and M270 are indicated, and the centers of these signals are marked with dots. (f). Crystal structure of A_2A_AR bound to LUF5833, where the E169–H264 salt bridge is disrupted (PDB code: 7ARO). The display style is identical to that in panels (b) and (c). (g) Overlaid ^1^H–^13^C HMQC spectra of A_2A_AR and A_2A_AR/H265A, labeled with [^2^H-8AA, αβγ-^2^H, methyl-^13^C-Met, α-^2^H, methyl-^13^C-Ala], bound to ZM241385. Only the regions containing A265 methyl resonances are shown.

In the A_2A_AR structures, ligands are bound at the base of a cavity in the extracellular region. Over the ligand binding site of A_2A_AR, a lid is formed by a salt bridge between E169 and H264, located at the intersection of the binding cavity and the extracellular loops (Fig. 1a). T256 forms a hydrogen bond network with E169 and H264.^34^ Upon disruption of this triad by E169Q or T256A mutations, the residence times of the ligand reportedly decrease by 62- and 17-fold, respectively, indicating that these residues regulate residence time.^34^ However, the mechanism by which residence time is regulated remains unclear due to the lack of structural information on these mutants. Whereas the E169–H264 salt bridge remains intact in the crystal structure of the A_2A_AR bound to a ligand, LUF5834, it is disrupted in the crystal structure of A_2A_AR bound to LUF5833, a derivative of LUF5834 with similar residence time.^17,35,36^ Therefore, these crystal structures alone cannot fully explain the role of the E169–H264 salt bridge in determining the residence time.

In this study, we observed the NMR signals of the methionine and alanine residues in the ligand binding site of A_2A_AR under conditions with varying residence times to identify conformational features related to residence time. Our findings indicate that the spatial arrangement surrounding the E169–H264 salt bridge correlates with residence time.

## Results

There are six methionine residues in A_2A_AR, including M174 and M270, which are located in the extracellular region (Fig. 1b). We previously observed and assigned the NMR signals of the methyl groups of these residues in the state bound with ZM241385, which reportedly has a residence time of 84 min.^28,34^ Additionally, A_2A_AR contains 32 alanine residues, with A165 and A265 positioned near the triad of E169, T256, and H264 (Fig. 1c). Therefore, we used the methionine and alanine methyl groups to investigate the conformation at the intersection of the binding cavity and the extracellular loops of A_2A_AR. The preparation of deuterated, alanine-, and methionine-selectively labeled A_2A_AR was achieved using the insect cell-baculovirus expression system, as previously reported.^37^ Deuterated amino acids were selected based on previous reports on labeling efficiencies^38^ and the ^1^H–^1^H distances between the observed methyl groups and other amino acid residues in the crystal structures of A_2A_AR. Our calculations revealed that, in the case of deuteration of isoleucine, leucine, valine, phenylalanine, threonine, lysine, arginine, proline, methionine (Hα, Hβ, and Hγ), and alanine (Hα), the ^1^H–^1^H dipole–dipole interactions of M174, M270, A165, and A265 would be reduced to 20–50% of those in non-deuterated A_2A_AR. Hereafter, the A_2A_AR obtained by this method is referred to as [^2^H-8AA, αβγ-^2^H, methyl-^13^C-Met, α-^2^H, methyl-^13^C-Ala] A_2A_AR.

In the ^1^H–^13^C HMQC spectra of [^2^H-8AA, αβγ-^2^H, methyl-^13^C-Met, α-^2^H, methyl-^13^C-Ala]A_2A_AR bound to ZM241385, the signals corresponding to approximately 20 alanine residues, along with previously assigned resonances from M140, M174, M211, and M270, were observed (Fig. 1d and e). To assign these resonances, spectra of the A165T and A265S mutants were recorded. As a result, one resonance was absent in the spectra of the A165T and A265S mutants, revealing that these resonances originate from A165 and A265 (Fig. S1). Assignment of the resonances from M270, A165, and A265 in the NECA-bound state was performed similarly (Fig. S2).

In the crystal structure of A_2A_AR bound to LUF5833, which has a shorter residence time than ZM241385,^36^ the E169–H264 salt bridge is disrupted by the imidazole moiety of LUF5833, causing the H264 sidechain oriented toward the extracellular surface (Fig. 1f).^35^ This structure suggests that the orientation of the H264 sidechain reflects the presence or absence of the E169–H264 salt bridge. The H264 sidechain induces a ring current effect on the neighbouring A265 methyl group (Fig. 1c). Upon H264A mutation, the resonance from the A265 methyl group exhibited an upfield ^1^H shift (Fig. 1g and S3). Thus, the ^1^H chemical shift of A265 reflects the presence or absence of the E169–H264 salt bridge. The aromatic rings of LUF5833 are located more than 8 Å away from the Cβ atom of A265 in the crystal structure. The calculated ring current shift is less than 0.01 ppm, indicating a negligible contribution.

**Fig. 2:**
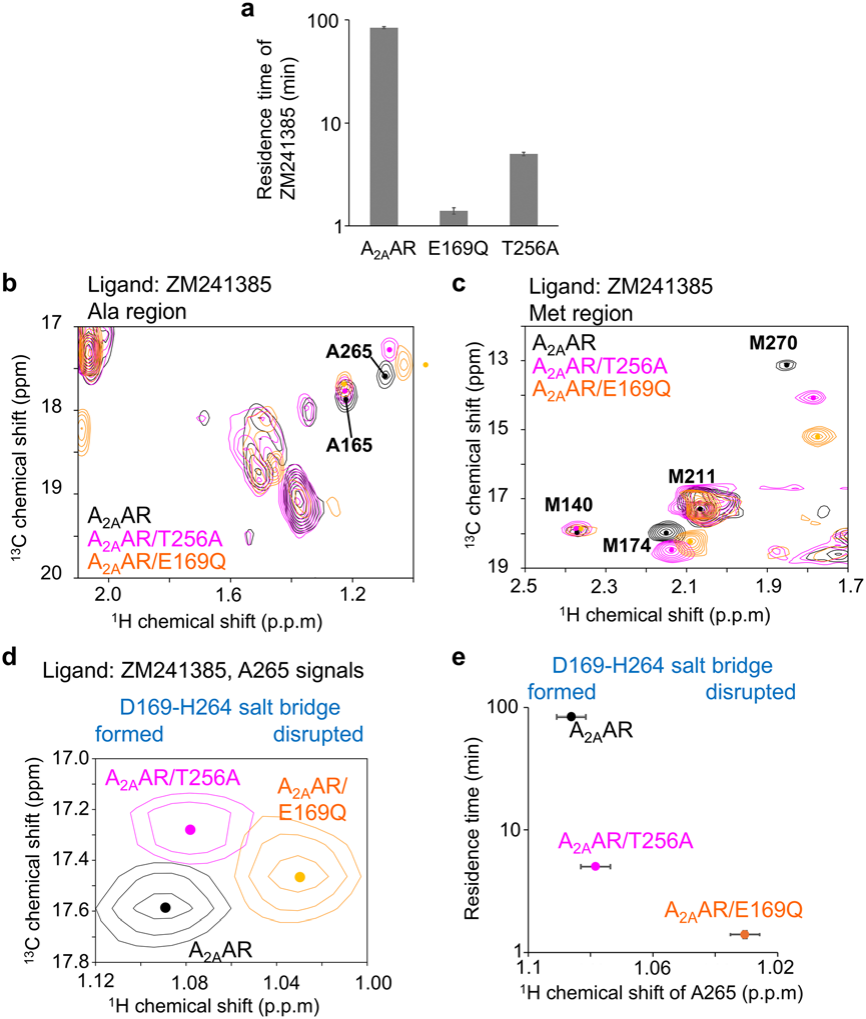
Conformation of A_2A_AR mutants with reduced ligand residence time. (a) Plot of the previously reported residence time of ZM241385 bound to A_2A_AR, A_2A_AR/E169Q, and A_2A_AR/T256A.^34^ (b)–(d). Alanine and methionine methyl regions of the overlaid ^1^H–^13^C HMQC spectra of A_2AA_R, A_2A_AR/T256A, and A_2A_AR/E169Q, labeled with [^2^H-8AA, αβγ-^2^H, methyl-^13^C-Met, α-^2^H, methyl-^13^C-Ala], bound to ZM241385. Only the regions containing A265 methyl resonances are shown in panel (d). (e) Plot of the ^1^H chemical shifts of the A265 methyl signals against the residence times in the ZM241385-bound state. The error values of the ^1^H chemical shifts were calculated from the digital resolutions.

To investigate the conformation of A_2A_AR under conditions with various residence times, we recorded the ^1^H–^13^C HMQC spectra of the E169Q and T256A mutants. In the A_2A_AR/E169Q mutant, the E169–H264 salt bridge was directly affected by the mutation, and the residence time was 62-fold lower than that of A_2A_AR^34^ (Fig. 2a). Chemical shifts of the resonances from M174, M270, and A265 were significantly different from those of the A_2A_AR/E169Q spectrum bound to ZM241385 (Fig. 2b–d and S4). These residues are located near the E169, H264, and T256 triad (Fig. 1c).

The ^1^H chemical shift of A265 in the E169Q mutant showed a significant upfield shift, similar to the shift seen in the H264A mutant (Fig. 1g and 2d). These results suggest that the E169–H264 salt bridge is disrupted in A_2A_AR/E169Q. In the spectrum of A_2A_AR/T256A, where the residence time is ∼17-fold lower than that of A_2A_AR, the ^1^H chemical shift of the A265 methyl group fell between those of the A_2A_AR/E169Q and A_2A_AR (Fig. 2b, d, and e), suggesting that the conformation surrounding the E169–H264 salt bridge is partially altered by the T256A mutation.

To examine the effects of these mutations in the presence of other ligands, we recorded spectra of A_2A_AR and its mutants in the state bound with NECA, which has a residence time slightly shorter than that of ZM241385 (35 minutes).^36^ In the spectra of A_2A_AR/T256A bound to NECA, the chemical shift of the A265 signal was also intermediate between those of A_2A_AR/E169Q and A_2A_AR (Fig. S5), suggesting that the E169Q and T256 mutations have similar effects on A_2A_AR in the NECA-bound state.

**Fig. 3:**
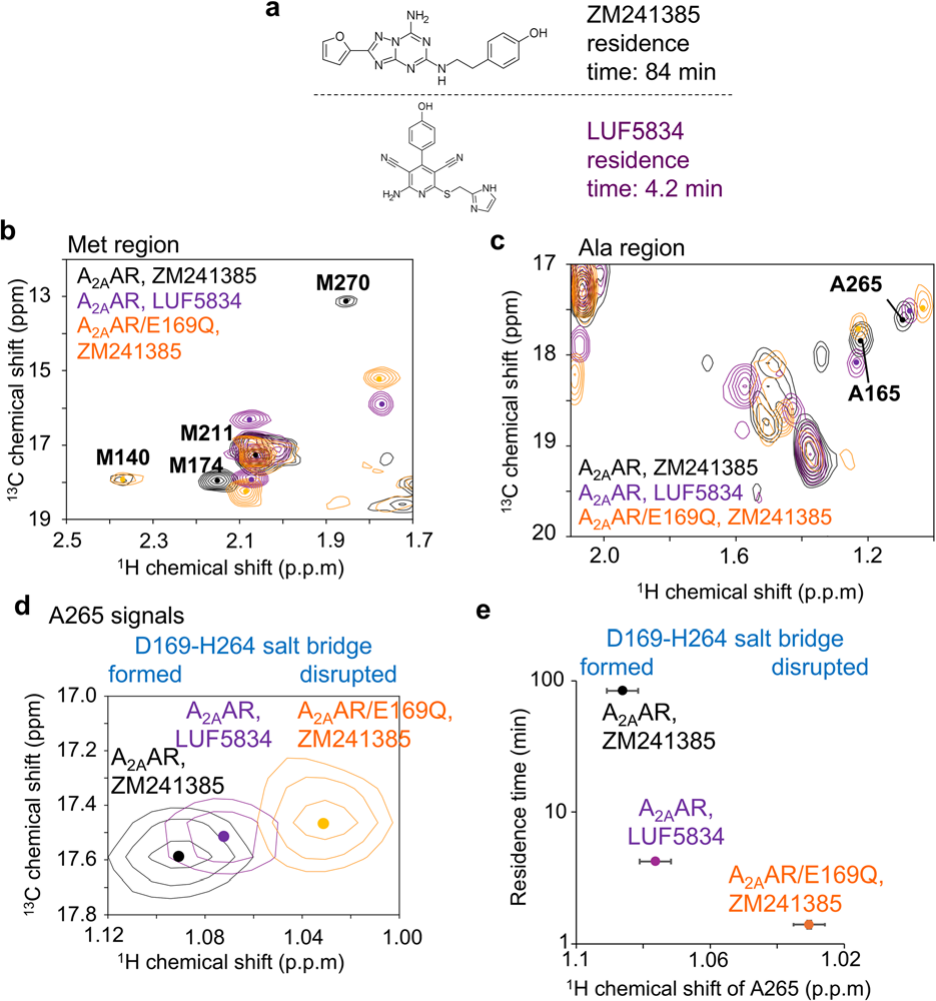
Conformation of the A_2A_AR bound to a ligand with a short residence time. (a) Chemical structures of ZM241385 and LUF5834. Residence time of LUF5834 bound to A_2A_AR was reported previously.^36^ (b)–(d). Alanine and methionine methyl regions of the overlaid ^1^H–^13^C HMQC spectra of A_2A_AR bound to ZM241385 (black), A_2A_AR bound to LUF5834 (purple), and A_2A_AR/E169Q bound to ZM241385 (orange). Only the region containing A265 methyl resonances is shown in panel (d). (e) Plot of the ^1^H chemical shifts of the A265 methyl signals against the residence times. The error values of the ^1^H chemical shifts were calculated based on the digital resolutions.

To further investigate the relationship between residence time and the conformation of the ligand binding site in A_2A_AR, we recorded resonances from methionine and alanine methyl groups of A_2A_AR bound to LUF5834, which reportedly has a residence time 19-fold shorter than that of ZM241385 (ref. 36) (Fig. 3a). The ^1^H chemical shift of A265 fell between those of A_2A_AR/E169Q and A_2A_AR in the ZM241385-bound state (Fig. 3b–e), indicating that the conformation surrounding the E169–H264 salt bridge is partially perturbed in the LUF5834-bound state.

To evaluate the dynamic behaviour of the E169–H264 salt bridge, we performed molecular dynamics (MD) simulations of A_2A_AR, A_2A_AR/T256A, and A_2A_AR/E169Q in the ZM241385-bound state. Root mean square distance (RMSD) analyses of the triad residues—E(Q)169, T(A)256, and H264—calculated with reference to the initial A_2A_AR structure (PDB ID: 4EIY), revealed that the RMSDs for all triad residues in A_2A_AR and A_2A_AR/T256A remained within the range of 0.6–0.9 Å. In contrast, A_2A_AR/E169Q exhibited significantly larger RMSDs: 1.11 ± 0.33 Å for Q169 and 1.49 ± 0.18 Å for H264 (Table S1). In addition, the average distance between E(Q)169 and H264 revealed that A_2A_AR had the shortest average distance (2.61 ± 0.57 Å), while A_2A_AR/E169Q had the longest (7.27 ± 1.52 Å) (Fig. S7). The results of the fragment molecular orbital (FMO) calculations^39,40^ based on the representative structures from MD simulations are shown in Fig. S8. In the two complexes, A_2A_AR and A_2A_AR/T256A, the electrostatic (ES) energy between E169 and H264 was below −100.0 kcal mol^−1^, indicating a strong electrostatic interaction and the presence of a salt bridge. In contrast, in A_2A_AR/E169Q, the ES energy between Q169 and H264 was −1.2 kcal mol^−1^, suggesting a weak electrostatic interaction and the lack of a salt bridge.

## Discussion

**Fig. 4:**
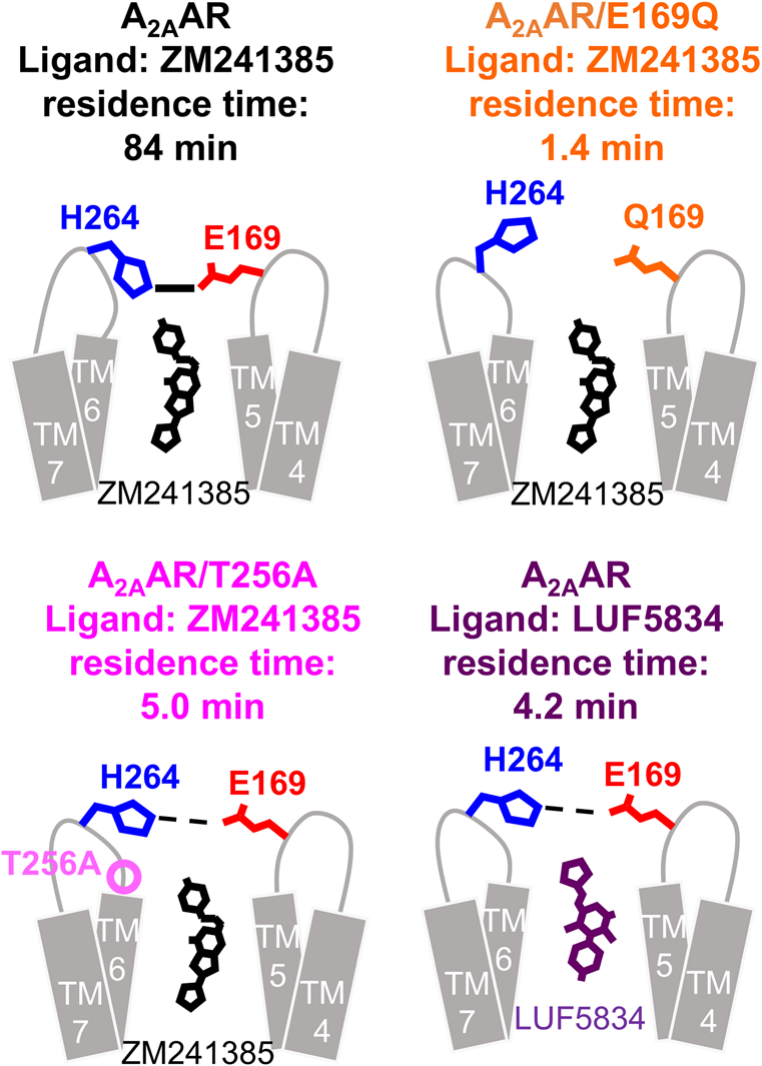
Schematic diagrams of the conformation surrounding E169–H264 salt bridge in A_2A_AR, related to ligand residence time. In A_2A_AR/E169Q, where the lowest residence time was observed, the E169–H264 salt bridge is broken. In A_2A_AR/T256A and A_2A_AR bound to LUF5834, both of which exhibited intermediate residence time, the conformation surrounding the E169–H264 salt bridge is partially perturbed.

Our NMR study of A_2A_AR-ligand complexes, which involved various types of mutations and ligands exhibiting up to a 60-fold difference in residence time, revealed that the ^1^H chemical shifts of A265, located near the lid on the ligand binding cavity formed by the E169–H264 salt bridge (Fig. 1a and f), strongly correlate with the residence time of the complex (Fig. 2 and 3). The ^1^H chemical shift of A265 is influenced by the ring current shift induced by the neighbouring aromatic group of H264 (Fig. 1c and g), and the crystal structures suggest that the orientation of the H264 side chain reflects the presence or absence of the E169–H264 salt bridge (Fig. 1f). Therefore, we conclude that the E169–H264 salt bridge and its surrounding environment modulate the residence time (Fig. 4). Molecular dynamics simulation visualized the experimentally observed behaviour of the E169–H264 salt bridge (Fig. S6, S7 and Table S1). The salt bridges observed in A_2A_AR and A_2A_AR/T256A were not formed in A_2A_AR/E169Q. In addition, the triad IFIE among E(Q)169–T(A)256–H264 was −6.0 kcal mol^−1^, which was significantly reduced compared to A_2A_AR and A_2A_AR/T256A (−115.5 and −112.5 kcal mol^−1^, respectively). Although multiple interactions between A_2A_AR and the ligands also contribute to the residence time, the conformation surrounding the E169–H264 salt bridge can account for up to a 60-fold difference in residence time.

Several crystal structures lacking the E169–H264 salt bridge have been reported (*e.g.* PDB ID 3PWH, 5OLO, and 3QAK).^15,41,42^ These absences have been attributed to the deprotonation of H264 at high pH or steric interactions between bulky ligands and E169/H264.^17,43^ However, the E169–H264 salt bridge is generally preserved under the pH and ligand conditions employed in this study, as described below. All NMR measurements in this study were conducted in 20 mM phosphate buffer at pH 7.0. Notably, A_2A_ARs crystal structures that retain the E169–H264 salt bridge were solved at pH ≥ 7.0 (*e.g.*, PDB IDs: 4UG2 and 2YDV).^44,45^ Furthermore, crystal structures that maintain the E169–H264 salt bridge have been reported for A_2A_AR bound to the ligands used in this study: ZM241385, NECA, and LUF5834 (*e.g.* PDB ID 4EIY, 2YDV, and 8RLN).^17,45,46^ Therefore, we conclude that the salt bridge is formed under our experimental conditions, and that its perturbation by E169Q and T256A mutations is physiologically relevant.

The transmembrane region of A_2A_AR exists in an equilibrium between inactive and active conformations, which determines efficacy, as illustrated by the correlation between efficacy and the chemical shifts of the resonances from M211 (Fig. S6A).^28^ These M211 chemical shifts were unaffected by the E169Q and T256A mutations (Fig. S6B and S6C). Furthermore, a correlation between residence times and the ^1^H chemical shifts of the resonances from A265 and M270 was observed in both the inverse agonist (ZM241385)- and full agonist (NECA)-bound states (Fig. 2 and S5). Thus, the conformation surrounding the E169–H264 salt bridge does not appear to influence the equilibrium between inactive and active conformations in the transmembrane region. However, the residence time of A_2A_AR ligands is reported to determine the duration of sustained agonist responses.^11^ Consequently, the conformation surrounding the E169–H264 salt bridge likely regulates signaling activity in a manner distinct from that of the transmembrane region. In addition, these results suggest that the E169Q and T256A mutations do not perturb the A_2A_AR-ligand interactions responsible for regulating signalling activity. Crystal structures of A_2A_AR indicates that agonists activate A_2A_AR through the interactions with V84, T88, S277 and H278, located at the bottom of the ligand-binding cavity in TM3 and TM7.^45^ These interactions are unlikely to be affected by the E169Q and T256A mutations.

In the LUF5834-bound state, the chemical shift of the resonance from M211 lies between those observed in the ZM241385 (inverse agonist)-bound state and NECA (full agonist)-bound states (Fig. S6A), consistent with the classification of LUF5834 as a partial agonist. Therefore, the difference in residence time between ZM241385 and LUF5834 may be influenced not only by the conformation around the E169–H264 salt bridge but also by interactions with residues at the bottom of the ligand-binding cavity. In the crystal structure of the A_2A_AR-LUF5834 complex, the E169–H264 salt bridge is intact, similar to those seen in the A_2A_AR-ZM241385 and A_2A_AR-NECA complexes.^17^ In contrast, in the crystal structure of A_2A_AR bound to LUF5833, a derivative of LUF5834 that lacks a phenolic hydroxyl group, the E169–H264 salt bridge is disrupted by the imidazole moiety of LUF5833 (Fig. 1f).^35^ It is unlikely that the phenolic hydroxyl group directly affects the structure surrounding the E169–H264 salt bridge, as it is located on the opposite side of the ligand-binding site. This leads us to speculate that in the LUF5834-bound state, A_2A_AR exists in equilibrium between conformations similar to those observed in these crystal structures, resulting in an intermediate residence time. The thermodynamic equilibrium dissociation constants reportedly changed by only 2.3-fold and 2.5-fold due to the E169Q and T256A mutations, respectively, whereas the residence time increased by 62-fold and 17-fold (Fig. S9).^34^ This suggests that the E169–H264 salt bridge slows both binding and dissociation kinetics.

We have observed NMR signals of methionine methyl groups and leucine mainchain amide groups of β_2_ adrenergic receptor and μ opioid receptor.^37,38,47,48^ However, in these studies, we did not observe conformational features related to ligand residence time. This may be due to the lack of observable signals from residues in the extracellular loops, as leucine and methionine residues are predominantly located in the transmembrane region. In the present study, by applying our alanine-selective labelling method using the insect-cell expression system,^49^ we were able to observe and assign resonances from alanine residues located in the extracellular loops, specifically A165 and A265.

Portions of the extracellular loops of other GPCRs are also proposed to function as lids that regulate ligand residence times. For example, in the crystal structure of the M3 muscarinic acetylcholine receptor, the ligand tiotropium is shielded by a cluster of tyrosine residues on the extracellular side, and molecular dynamics simulations suggest that the residence time is related to the mobility of this extracellular region.^4,50^ In the β-adrenergic receptor, which has been extensively studied by NMR as well as A_2A_AR,^51–53^ the ligand-binding pocket is partially covered by a salt bridge formed by D192 and K305, which is thought to control ligand entry and exit pathways.^54^

Drugs targeting A_2A_AR have diverse applications; for instance, regadenoson is used for myocardial imaging, while istradefylline is prescribed for the treatment of Parkinson's disease.^9^ The optimal residence times for these drugs differ according to their clinical use; shorter residence times are preferable for the former, while longer residence times are desirable for the latter. Monitoring the signals of A265 and M270 may prove useful in the development of drugs targeting A_2A_AR, with residence times tailored to achieve the desired pharmacological effects.

## Conclusions

Our studies on A_2A_AR under various ligand residence time conditions revealed that the presence or absence of the E169–H264 salt bridge correlates with the ligand residence time. These findings offer valuable insights into the ligand dissociation pathway and may aid in the development of drugs targeting A_2A_AR and other GPCRs with tailored residence times.

## Author contributions

Conceptualization: T. U., Y. K., K. T., I. S. Methodology: T. U., T. T., M. K., T. M., Y. K., S. M., K. F., S. I., Y. S. Investigation: T. U., T. T., M. K., T. M., Y. K., S. M., K. F. Visualization: T. U., T. T. Supervision: T. U., K. T., I. S. Writing—original draft: T. T., T. U., Y. K. Writing—review & editing: T. U., S. I., Y. S., K. T., I. S.

## Conflicts of interest

There are no conflicts to declare.

## Supplementary Material

SC-OLF-D5SC02398J-s001

## Data Availability

The FMO calculation results were deposited in FMODB (https://drugdesign.riken.jp/FMODB/),^55^ and the FMODBIDs are NZ23Q (A_2A_AR), G8YQ1 (A_2A_AR/T256A) and 8GNJY (A_2A_AR/E169Q). The data supporting this article have been included as part of the SI: Materials and methods; Fig. S1: Assignment of the resonances from A165 (A) and A265 (B) of A__2A__AR bound to ZM241385; Fig. S2: Assignment of the resonances from alanine and methionine residues in A__2A__AR bound to NECA; Fig. S3: NMR spectra of A__2A__AR/H264A; Fig. S4: Normalized chemicals shift differences of methionine and alanine methyl resonances between A__2A__AR and A__2A__AR/H264A in the ZM241385-bound state; Fig. S5: Conformation of the A__2A__AR mutants with reduced ligand residence time, in the NECA-bound state; Fig. S6: Effect of the T256A and E169Q mutations on the equilibrium between active and inactive conformations of A__2A__AR; Fig. S7: Representative structures of MD simulations; Fig. S8: Interaction energy analysis by FMO calculations; Fig. S9: Plot of the previously reported equilibrium dissociation constants of ZM241385 bound to A__2A__AR, A__2A__AR/E169Q, and A__2A__AR/T256A; Table S1: Root mean square deviation (RMSD) analysis of ZM241385, E(Q)169, H264, and T(A)256 in the A__2A__AR, A__2A__AR/E169Q, and A__2A__AR/T256A structures; references. See DOI: https://doi.org/10.1039/d5sc05477j.
