# Supplementary material for: Structural basis of the residence time of adenosine A2A receptor ligands revealed by NMR
Source: Chem Sci. 2025 Aug 29;16(38):17948–55. doi: 10.1039/d5sc02398j (PMC12416388; doi:10.1039/d5sc02398j)
Supplement: SC-016-D5SC02398J-s001 [file SC-016-D5SC02398J-s001.pdf]

Supplementary Information (SI) for Chemical Science.
This journal is © The Royal Society of Chemistry 2025

Supporting Information for

Structural basis of the residence time of adenosine A_{2A} receptor ligands revealed by NMR

Takumi Ueda[a,b], Tomoki Tsuchida[a], Masatoshi Kurita[a], Takuya Mizumura[a], Shunsuke Imai[a,c], Yutaro Shiraishi[a,b,c], Yutaka Kofuku[a], Shuhei Miyakawa[b], Kaori Fukuzawa[b], Koh Takeuchi[b], Ichio Shimada*[a,c,d]

[a]Graduate School of Pharmaceutical Sciences, The University of Tokyo, Hongo, Bunkyo-ku, Tokyo 113-0033, Japan

[b]Graduate School of Pharmaceutical Sciences, Osaka University, 1-6, Yamadaoka, Suita-Shi, Osaka 565-0871 Japan

[c]Center for Biosystems Dynamics Research, RIKEN, 1-7-22, Suehiro-cho, Tsurumi-ku, Yokohama, Kanagawa 230-0045, Japan

[d]Graduate School of Integrated Sciences for Life, Hiroshima University, 1-4-4 Kagamiyama, Higashi-Hiroshima City, Hiroshima 739-8528, Japan

*To whom correspondence should be addressed. E-mail. ichio.shimada@riken.jp

Table of Contents

Materials and Methods

Figure S1. Assignment of the resonances from A165 (A) and A265 (B) of $A_{2A}AR$ bound to ZM241385.

Figure S2. Assignment of the resonances from alanine and methionine residues in $A_{2A}AR$ bound to NECA.

Figure S3. NMR spectra of $A_{2A}AR$/H264A.

Figure S4. Normalized chemicals shift differences of methionine and alanine methyl resonances between $A_{2A}AR$ and $A_{2A}AR$/H264A in the ZM241385-bound state.

Figure. S5. Conformation of the $A_{2A}AR$ mutants with reduced ligand residence time, in the NECA-bound state.

Figure. S6. Effect of the T256A and E169Q mutations on the equilibrium between active and inactive conformations of $A_{2A}AR$.

Figure. S7. Representative structures of MD simulations.

Figure. S8. Interaction energy analysis by FMO calculations.

Figure. S9. Plot of the previously reported equilibrium dissociation constants of ZM241385 bound to $A_{2A}AR$, $A_{2A}AR$/E169Q, and $A_{2A}AR$/T256A.

Table. S1. Root mean square deviation (RMSD) analysis of ZM241385, E(Q)169, H264, and T(A)256 in the $A_{2A}AR$, $A_{2A}AR$/E169Q, and $A_{2A}AR$/T256A structures.

References

Materials & Methods

Reagents

All reagents were purchased from Wako Chemicals or Nacalai Tesque unless otherwise noted. The [α,β,β-^{2}H, methyl-^{13}C]-L-methionine was synthesized by the enzymatic deuteration of [methyl-^{13}C]-L-methionine (ISOTEC or Cambridge Isotope Laboratories) with *Escherichia coli* cystathionine-γ-synthase, as previously described[1]. The XAC-agarose gel was prepared as reported previously[2]. Amino acid deficient ESF921 medium with polished yeast extract 2 (ESF921 ΔAA) was purchased from Expression Systems (Catalogue Number 96-275). L-aspartic acid sodium salt, L-glutamic acid sodium salt, L-asparagine, and L-glutamine were dissolved at 6, 8, 6, and 12 mg/mL, respectively, in ESF921 ΔAA (4 × DENQ stock). The [^{2}H]-algal amino acid mixture (ISOTEC) was dissolved at 25 mg/mL in H_2O with gentle heating and ultrafiltrated with a Centriconplus-70 (10 kDa molecular weight cut off, Millipore). L-tyrosine disodium salt hydrate (Sigma-Aldrich) was dissolved at 8 mg/mL in H_2O. Unlabeled L-cystine dihydrochloride was dissolved at 5.2 mg/mL in 100 mM HCl. Other amino acids were individually dissolved at 10-100 mg/mL in H_2O.

The cDNA fragment encoding human A_{2A}AR(1-316) with N-terminal FLAG tag and a C-terminal 10x His-tag was amplified by PCR and cloned into the pFastBac1 vector (Invitrogen) via the BamHI-HindIII sites. Mutations were introduced using either a QuikChange® site-directed mutagenesis kit or a QuikChange® multi site-directed mutagenesis kit (Stratagene). M1A, M4T, F93W, N154Q, A221T, and A316S mutations were introduced in all constructs, unless otherwise stated.

Sf9 cells (Invitrogen), utilized for the production of recombinant baculoviruses, were routinely maintained at 27 °C in Grace's supplemented medium (GIBCO) containing 10% fetal bovine

serum (Biowest), 0.1% Pluronic F-58 (GIBCO), 50 international units/mL penicillin, 50 mg/mL streptomycin, and 0.125 mg/mL amphotericin B (GIBCO, as Antibiotic-Antimycotic). Recombinant baculoviruses were generated and amplified with the Bac-to-Bac system (Invitrogen), according to the manufacturer's instructions.

Expression and purification of $A_{2A}AR$

The expresSF+® cells (SF+ cells, Protein Sciences Corp.), used for the expression of A_{2A}AR, were maintained as previously described[1]. For the expression of [(^{2}H-8AA, α,β,γ-^{2}H, ε-^{13}C)-Met, (α-^{2}H-, methyl-^{13}C)-Ala] A_{2A}AR, 75 mL of 4 × DEQN stock, 9 mL of 1 M NaCl, 3 mL of 50 mg/mL glycine, 3 mL of 50 mg/mL serine, 3 mL of 10 mg/mL tryptophan, 3 mL of 30 mg/mL histidine, 9 mL of 5.2 mg/mL DL-cystine, 1 mL of 8 mg/mL L-tyrosine disodium salt hydrate, 0.3 mL of 10 mg/mL β-chloro-L-alanine-HCl, and 1.5 mL of 10 mg/mL [α, β, γ-^{2}H, ε-^{13}C] L-methionine were added to 141 mL of ESF921 ΔAA. SF+ cells in Sf-900 II medium (GIBCO) were centrifuged at 200×g and resuspended in the above medium at approximately 2 × 10^6 cells/mL. The cells were inoculated with 3-4 mL of the high-titer virus stock and maintained at 27 °C. At 20 hours post-infection, 6 mL of 50 mg/mL [α-^{2}H, methyl-^{13}C]-alanine, 3 mL of 10 mg/mL [α, β, γ-^{2}H, ε-^{13}C] L-methionine, 0.3 mL of 5 mg/mL E-64, 1.5 mL of 10 mg/mL [^{2}H]-phenylalanine (CIL), 1.5 mL of 10 mg/mL [^{2}H]-valine (CIL), and 0.3 mL of 10 mg/mL β-chloro-L-alanine hydrochloride (SIGMA) were added. The cells were harvested at 48 hr post-infection by centrifugation at 800 × g, and the resulting cell pellets were stored at -80 °C.

All subsequent procedures were either performed on ice or in a cold room (4 °C) unless otherwise noted. The pellet from 2.4 L of cell culture was resuspended in 320 mL of buffer A (50 mM Tris, pH 7.4, 200 mM NaCl, 1 mM EDTA, 0.5 mM 4-(2-aminoethyl) benzenesulfonyl fluoride

hydrochloride (AEBSF), 0.3 μM aprotinin (Wako Chemicals), 10 μM leupeptin hemisulfate (Peptide Institute), 15 μM pepstatin A (Peptide Institute), and 14 μM E-64). Cells were disrupted by nitrogen cavitation (Parr Bomb) at 600 p.s.i. for 30 min. The lysate was centrifuged at 800×g for 10 min, and the supernatant was centrifuged at 100,000×g for 60 min. The membrane pellet was resuspended by Dounce homogenization in 50 mM HEPES-NaOH, pH 7.4, 500 mM NaCl, 30% glycerol, 10 mM theophylline, 0.1 mM AEBSF, 14 μM E-64, 10 μM leupeptin, and was stored at -80 °C.

The membrane pellet obtained from 2.4 L of cell culture was solubilized in 80 mL of 20 mM HEPES-NaOH, pH 7.4, 500 mM NaCl, 2% n-dodecyl-β-D-maltopyranoside (DDM, Dojindo), 0.4 mM cholesteryl hemisuccinate (CHS), 10 mM theophylline, 0.1 mM AEBSF, 14 μM E-64 and 10 μM leupeptin) for 4 h, and were then centrifuged at 100,000×g for 1 h. The supernatant was batch incubated overnight with 2 mL of TALON metal affinity resin (Clontech). The resin was washed with 60 mL of buffer B (20 mM HEPES-NaOH, pH 7.4, 500 mM NaCl, 10% glycerol, 0.1% DDM, 0.02% CHS, 14 μM E-64, and 10 μM leupeptin) supplemented with 5 mM imidazole, and the protein was eluted with 12 mL of buffer B supplemented with 250 mM imidazole. The eluate was batch incubated for 5 h with 4 mL of XAC-agarose gel. The resin was washed with 120 mL of buffer B, and the protein was eluted with 88 mL of buffer B supplemented with 30 mM theophylline. The eluate was batch incubated for 1.5 h with 0.5 ml of TALON resin. The resin was washed with 10 ml of buffer B, supplemented with the ligand (100 μM NECA, 25 μM UK432097, 50 μM CGS21680, 100 μM ZM241385, or 50 μM LUF5834), and the protein was eluted with 1 ml of buffer B, supplemented with 200 mM imidazole and the ligand. The eluate from the second TALON affinity step was concentrated using a centrifugal filter device (AmiconUltra-4, 30 kDa molecular weight cutoff, Millipore), while simultaneously exchanging

the buffer to buffer C (20 mM sodium phosphate, pH 7.0, 0.2 mM AEBSF, 28 μM E-64, 20 μM leupeptin, H_2O/D_2O = 1/99), supplemented with the ligand.

For the ligand exchange after the NMR measurement, 2.5 mL of A_{2A}AR solution in buffer C was loaded on PD-10 column (GE Healthcare), and eluted with 3.5 mL of buffer B. The eluate was batch incubated for 1.5 h with 0.5 mL TALON resin. The resin was washed with 10 ml of buffer B supplemented with the ligand, and the protein was eluted with 1 ml of buffer B, supplemented with 200 mM imidazole and the ligand. The eluate was concentrated, while simultaneously exchanging the buffer, as described above.

NMR experiments

The ^{1}H-^{13}C HMQC and one-dimensional ^{1}H spectra were recorded with a Bruker Avance 800 spectrometer equipped with a cryogenic probe. ^{1}H-^{13}C HMQC spectra were recorded for 15-40 μM [[α,β,β-^{2}H, methyl-^{13}C] Met] A_{2A}AR or its mutant in DDM micelles in buffer C. Spectral widths were set to 12,800 Hz and 6,400-7,400 Hz for the ^{1}H and ^{13}C dimensions, respectively, with the inter-scan delays of 1 sec. A total of 512×128 complex points were recorded, and 256 scans/FID gave rise to an acquisition times of 20–23 hours per spectrum. Spectra were referenced to 3-(trimethysilyl)-1-propanesulfonic acid sodium salt, in both dimensions. Data were processed and analyzed using Topspin software (versions 2.1, 3.1, and 3.5, Bruker).

Molecular dynamics simulation

The initial structures for MD simulations were prepared using the crystal structure of the A_{2A}AR –ZM241385 complex (PDB ID: 4EIY)[3]. The coordinates of A_{2A}AR, ZM241385, and crystallographic water molecules were extracted from the crystal structure. Missing residues and

hydrogen atoms were added, resulting in the H264 residue being doubly protonated. Then single-point mutations were introduced into $A_{2A}AR$ to generate the $A_{2A}AR$/E169Q and $A_{2A}AR$/T256A variants. Energy minimizations were performed on all structures with positional constraints applied to the heavy atoms of the modeled residues. These molecular modeling procedures were performed using Molecular Operating Environment version 2022.02[4].

All MD simulations were performed using GROMACS 2025.1[5]. Topology files were generated with AmberTools 24[6]. The AMBER ff14SB force field[7] was used for the $A_{2A}AR$ protein, and the general AMBER force field 2 (GAFF2)[8] was used for ZM241385 ligand. Atomic charges for ZM241385 were calculated by the restrained electrostatic potential (RESP) method at the HF/6-31G level using Gaussian 16[9]. Each system was neutralized with Cl^- counterions and solvated in a periodic box using the TIP3P water model. After energy minimization, the system was gradually heated from 100 K to 310 K over 1 ns under the NVT ensemble, followed by an additional 1 ns simulation at 310 K. Subsequently, density relaxation was performed under NPT ensemble (1.0 bar) for 1 ns. Finally, a 105 ns production run was conducted under NPT ensemble. Throughout all simulation steps, positional restraints with a force constant of 1,255 $kJ \cdot mol^{-1} \cdot nm^{-2}$ were applied to the backbone heavy atoms, while no constraints were applied to bond lengths. The time step was 0.5 fs for all simulations.

Fragment molecular orbital calculation

From the final 100 ns of the MD trajectory for each system, snapshots were extracted every 1 ns and subjected to clustering using the K-means algorithm. The number of clusters was set to five, and the representative structure from the largest cluster was selected for fragment molecular orbital (FMO) calculations[10, 11]. The theoretical level of the FMO calculation was FMO2-MP2[12, 13] with the 6-31G* basis set, and the calculations were performed using the ABINIT-MP

program[11]. The inter-fragment interaction energies (IFIE) were calculated from the FMO calculations, and were further decomposed by pair interaction energy decomposition analysis (PIEDA) into four energy components: electrostatic (ES), exchange repulsion (EX), charge transfer with mixed terms (CT), and dispersion (DI)[14]. Hydrogen bonds are primarily detected by stabilization energies in the ES and CT components, whereas hydrophobic interactions such as CH/π and π–π stacking are mainly detected by stabilization in the DI component[15]. The triad IFIE of E(Q)169–T(A)256–H264 was calculated by summing the individual IFIE values of E(Q)169–T(A)256, T(A)256–H264, and E(Q)169–H264.

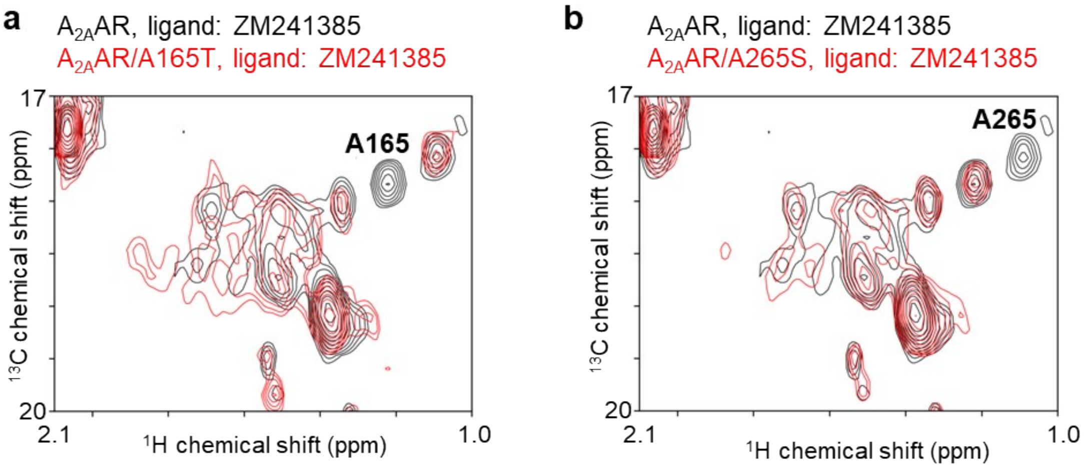

Fig. S1 Assignment of the resonances from A165 (a) and A265 (b) of A_{2A}AR bound to ZM241385.

Black and magenta spectra represent the ^{1}H-^{13}C HMQC spectra of [^{2}H-8AA, αβγ-^{2}H, methyl-^{13}C-Met, α-^{2}H, methyl-^{13}C-Ala] A_{2A}AR with and without the alanine residues to be assigned, respectively. The mutants used for the assignments are indicated at the top of each panel.

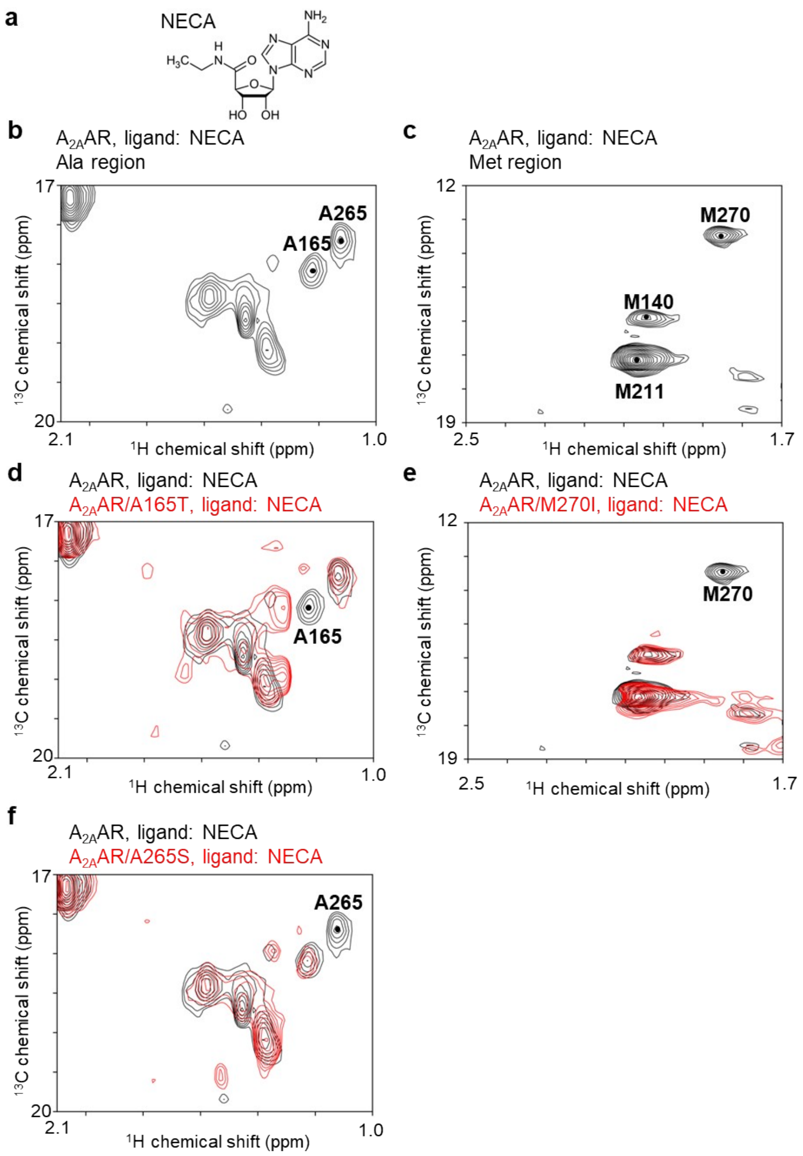

Fig. S2 Assignment of the resonances from alanine and methionine residues in A_{2A}AR bound to NECA. (a) Chemical structure of NECA. (b)-(c) ^{1}H-^{13}C HMQC spectra of [^{2}H-8AA, αβγ-^{2}H, methyl-^{13}C-Met, α-^{2}H, methyl-^{13}C-Ala] A_{2A}AR bound to NECA. Only the alanine and methionine methyl regions are shown in panels (b) and (c), respectively. The resonances from A165, A265, M140, M211, and M270 are indicated, with the centers of these signals marked with dots. (d)-(f). Assignment of the resonances from A165 (d), M270 (e) and A265 (f) in the presence of NECA. Black and magenta spectra represent the ^{1}H-^{13}C HMQC spectra of [^{2}H-8AA, αβγ-^{2}H, methyl-^{13}C-Met, α-^{2}H, methyl-^{13}C-Ala] A_{2A}AR with and without the alanine residues to be

assigned, respectively. The mutants used for the assignments are indicated at the top of each panel.

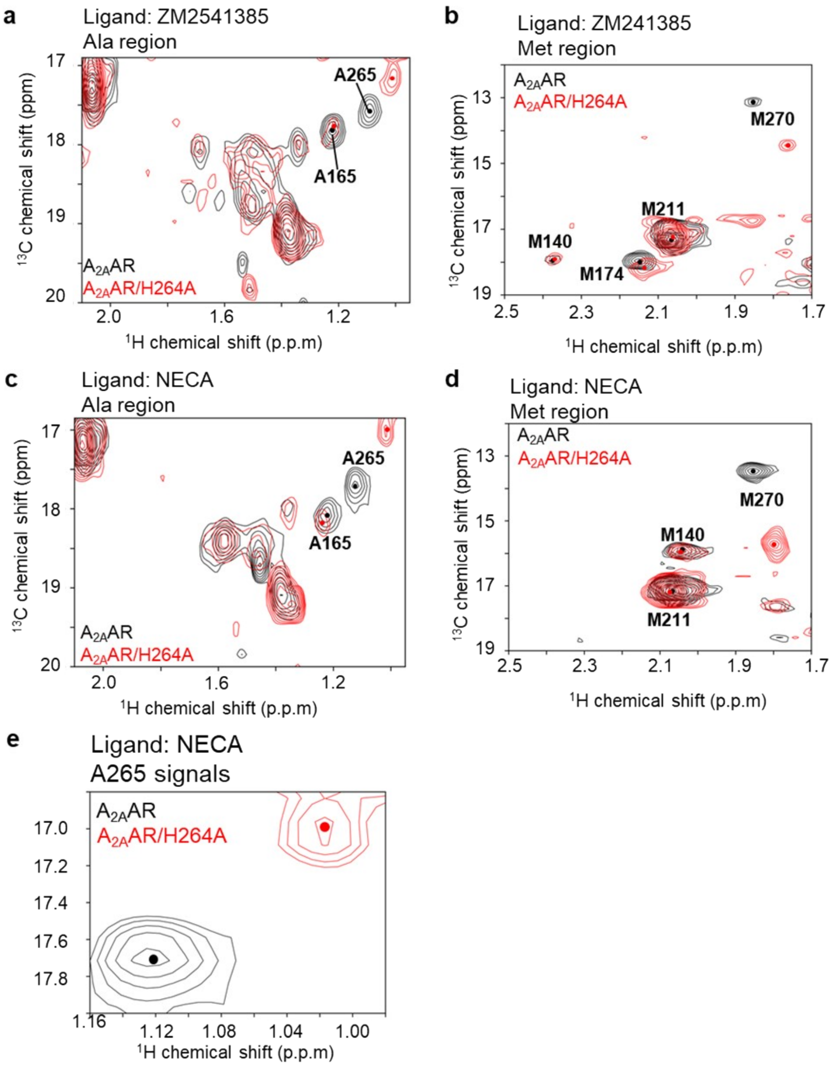

Fig. S3 NMR spectra of A_{2A}AR/H264A. (a)-(b) Overlaid ^{1}H-^{13}C HMQC spectra of A_{2A}AR and A_{2A}AR/H264A, labeled with [^{2}H-8AA, αβγ-^{2}H, methyl-^{13}C-Met, α-^{2}H, methyl-^{13}C-Ala], bound to ZM241385. Only the alanine and methionine methyl regions were shown in panels A and B, respectively. (c)-(e). Overlaid ^{1}H-^{13}C HMQC spectra of A_{2A}AR and A_{2A}AR/H264A, labeled with [^{2}H-8AA, αβγ-^{2}H, methyl-^{13}C-Met, α-^{2}H, methyl-^{13}C-Ala], bound to NECA. Only the alanine and methionine methyl regions were shown in panels (c) and (d), respectively, with the regions containing A265 methyl resonances shown in panel (e). The resonances from A165, A265, M140, M211, and M270 are indicated, with the centers of these signals marked with dots.

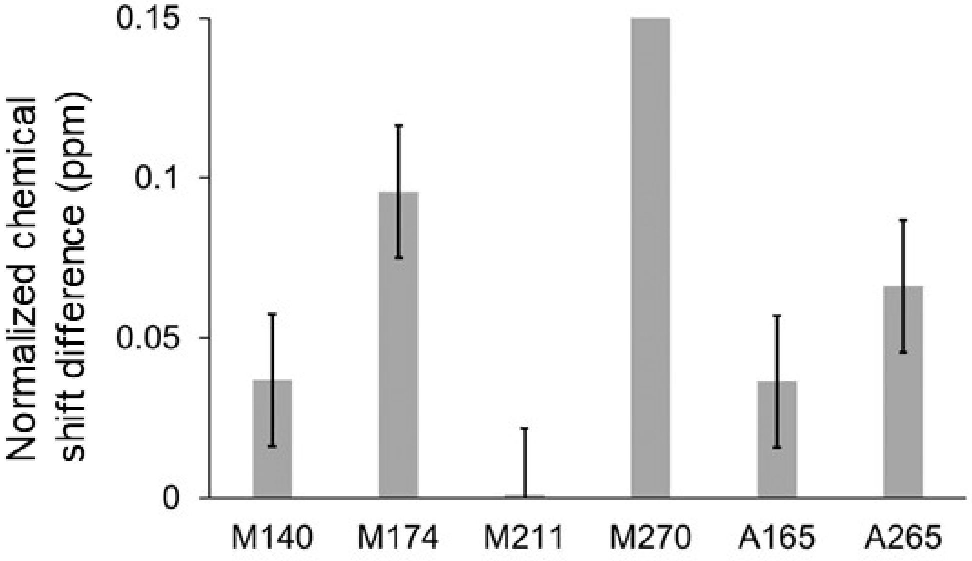

Fig. S4 Normalized chemicals shift differences of methionine and alanine methyl resonances between $A_{2A}AR$ and $A_{2A}AR$/H264A in the ZM241385-bound state.

Normalized chemical shift differences, $\Delta\delta$, were calculated using the equation $\Delta\delta=\{(\Delta\delta_{1H})^2+(\Delta\delta_{13C}/3.5)^2\}^{0.5}$. The normalization factor (3.5) is the ratio of the standard deviations of the methionine methyl ^{1}H and ^{13}C chemical shifts, as deposited in the Biological Magnetic Resonance Data Bank (http://www.bmrb.wisc.edu/). Error values were calculated using the formula $\{\Delta\delta_{1H}\cdot R_{1H}+\Delta\delta_{13C}\cdot R_{13C}/(3.5)^2\}/\Delta\delta$, where R_{1H} and R_{13C} are the digital resolutions in p.p.m. for the ^{1}H and ^{13}C dimensions, respectively.

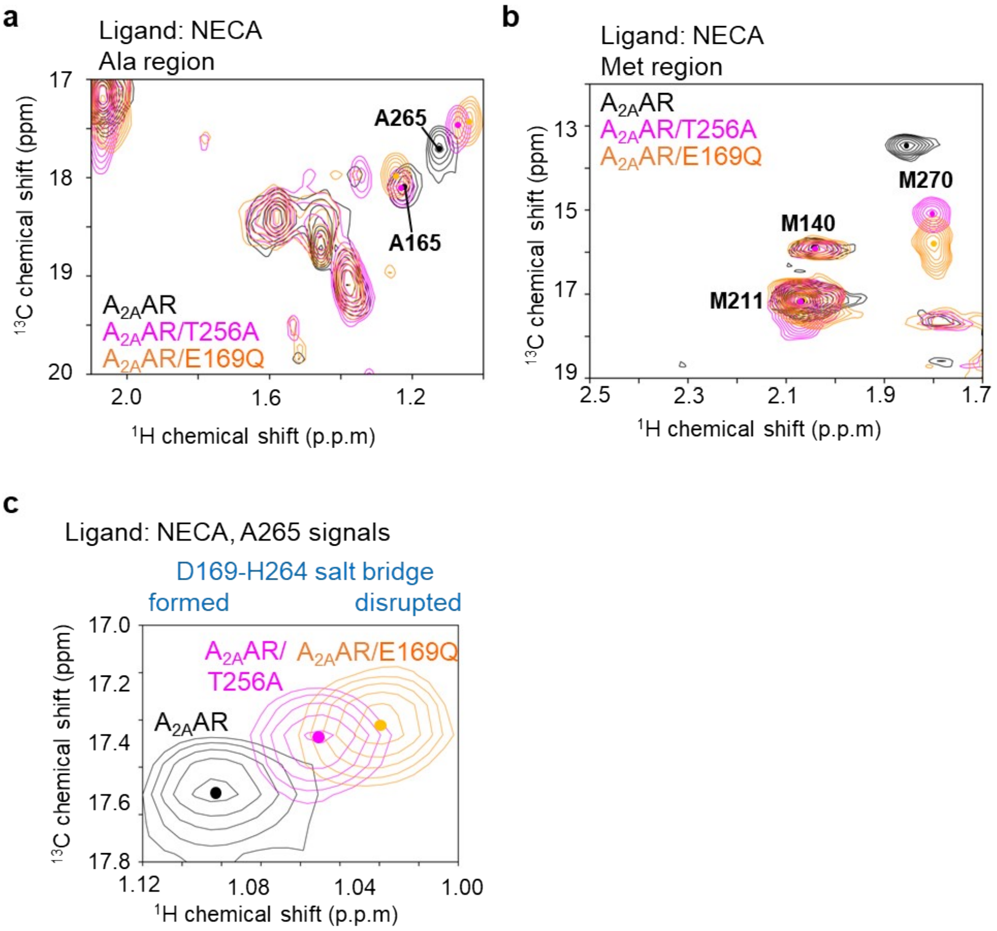

Fig. S5 Conformation of the $A_{2A}AR$ mutants with reduced ligand residence time, in the NECA-bound state. Alanine and methionine methyl regions of the overlaid ^{1}H-^{13}C HMQC spectra of $A_{2A}AR$, $A_{2A}AR$/T256A, and $A_{2A}AR$/E169Q, labeled with [^{2}H-8AA, αβγ-^{2}H, methyl-^{13}C-Met, α-^{2}H, methyl-^{13}C-Ala], bound to NECA, are shown in panels (a) and (b), respectively. Only the region with A265 methyl resonances is shown in (c). The relationship between the ^{1}H chemical shift of A265 and the condition of the D169-H264 salt bridge is illustrated on the top of the panel C.

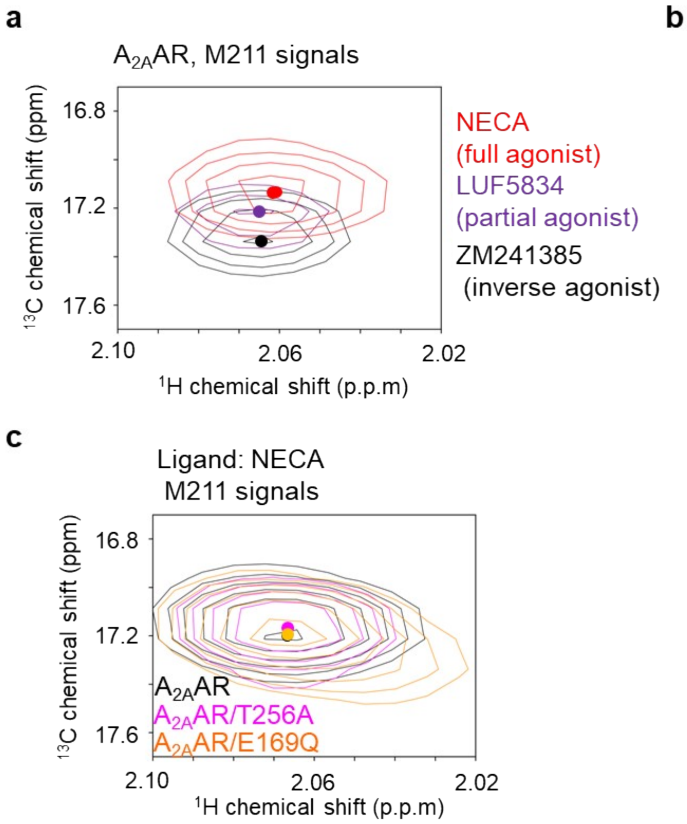

Fig. S6 Effect of the T256A and E169Q mutations on the equilibrium between active and inactive conformations of A_{2A}AR.

(a) Overlaid ^{1}H-^{13}C HMQC spectra of A_{2A}AR bound to an inverse agonist ZM241385 (black), a partial agonist LUF5834 (purple), and a full agonist NECA (red). (b)-(c) Overlaid ^{1}H-^{13}C HMQC spectra of A_{2A}AR, A_{2A}AR/T256A, and A_{2A}AR/E169Q, labeled with [^{2}H-8AA, αβγ-^{2}H, methyl-^{13}C-Met, α-^{2}H, methyl-^{13}C-Ala], bound to ZM241385 and NECA, are shown in panels (b) and (c), respectively. Only the regions containing M211 methyl resonances are shown, with the centers of the M211 signals indicated by dots.

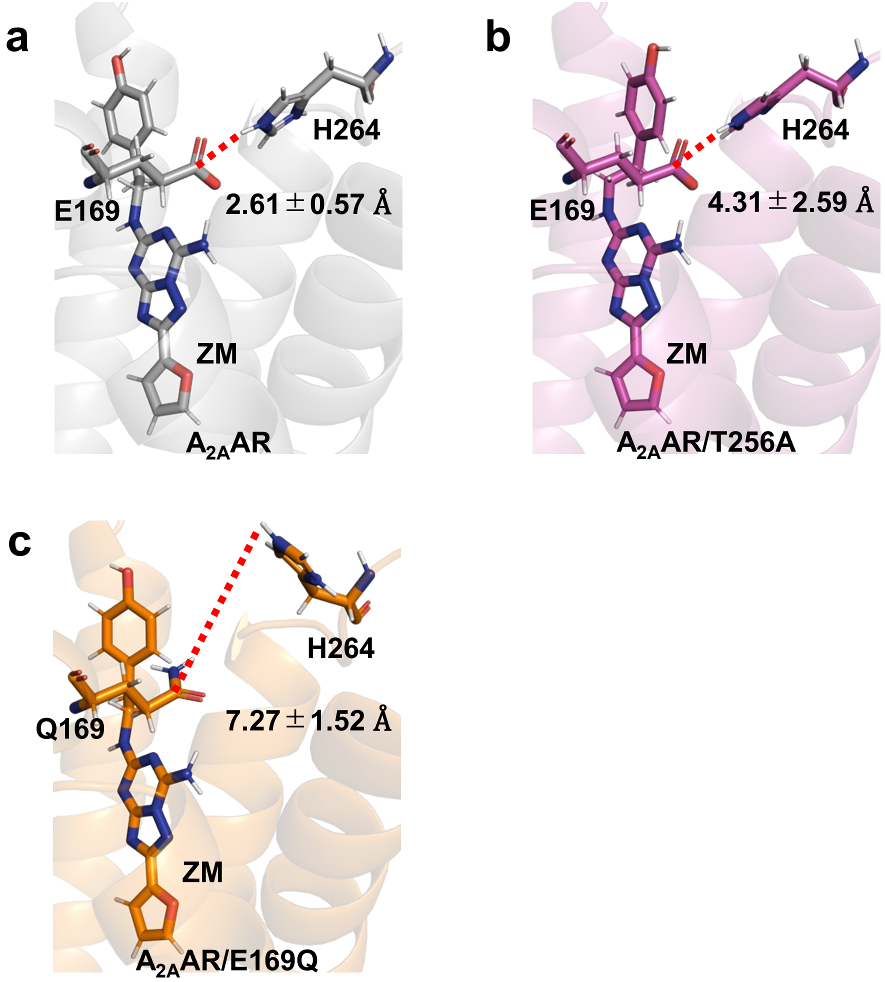

Fig. S7 Representative structures of MD simulations.

ZM241385, E(Q)169, and H264 were shown as stick models. The E(Q)-H264 distances were measured as the distance between the Cδ atom of E(Q)169 and the Hε atom of H264. (a), (b), and (c) represent $A_{2A}AR$, $A_{2A}AR$/E169Q, and $A_{2A}AR$/T256A structures, respectively.

a

b

c

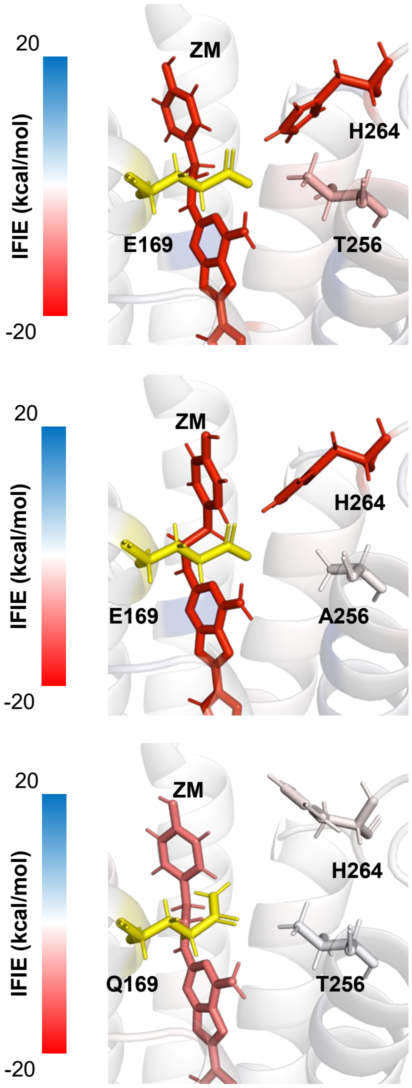

d

A_{2A}AR	ES	EX	CT	DI	IFIE
ZM binding energy	-64.3	59.9	-23.7	-51.7	-79.8
E169-T256	-5.2	0.0	-0.2	-0.2	-5.6
T256-H264	-0.3	0.1	-0.3	-0.7	-1.2
E169-H264	-108.8	8.3	-4.5	-3.6	-108.7

(kcal/mol)

e

A_{2A}AR/T256A	ES	EX	CT	DI	IFIE
ZM binding energy	-61.8	48.9	-22.2	-44.7	-79.7
E169-A256	-0.7	0.0	0.0	-0.2	-0.9
A256-H264	-0.2	0.0	-0.1	-0.4	-0.7
E169-H264	-113.9	12.7	-5.7	-3.9	-110.9

(kcal/mol)

f

A_{2A}AR/E169Q	ES	EX	CT	DI	IFIE
ZM binding energy	-55.1	51.5	-22.0	-51.3	-76.9
Q169-T256	1.1	0.0	-0.2	-0.3	0.6
T256-H264	-5.1	0.0	0.0	-0.3	-5.4
Q169-H264	-1.2	0.0	0.0	0.0	-1.2

(kcal/mol)

Fig. S8 Interaction energy analysis by FMO calculations.

Representative structures of (a) A_{2A}AR, (b) A_{2A}AR/T256A, and (c) A_{2A}AR/E169Q, with ZM241385 (ZM), E(Q)169, H264, and T(A)256 displayed by sticks. The IFIEs between E(Q)169 (highlighted in yellow) and surrounding residues are visualized by color. (d)–(f) show the IFIE and PIEDA for each structure. The ZM binding energy is the sum of the IFIE between ZM241385 and all amino acid residues. The FMO calculation results were deposited in FMODB (https://drugdesign.riken.jp/FMODB/)[16], and the FMODBIDs are NZ23Q (A_{2A}AR), G8YQ1 (A_{2A}AR/T256A) and 8GNJY (A_{2A}AR/E169Q).

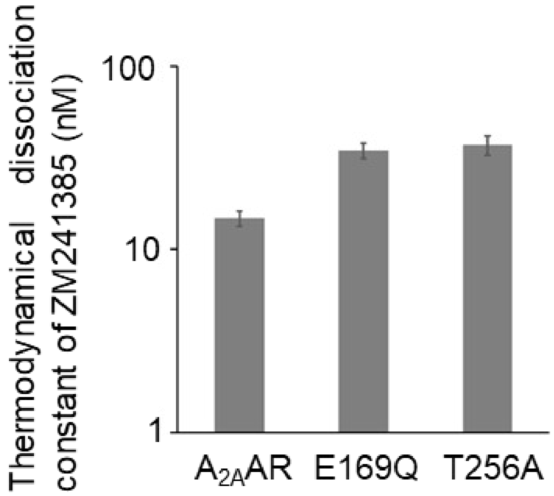

Fig. S9 Plot of the previously reported equilibrium dissociation constants of ZM241385 bound to A_{2A}AR, A_{2A}AR/E169Q, and A_{2A}AR/T256A[17].

RMSD (Å)	ZM	E(Q)169	H264	T(A)256
**A_{2A}AR**	1.54±0.40	0.58±0.40	0.58±0.50	0.68±0.26
**A_{2A}AR/T256A**	1.40±0.49	0.70±0.37	0.91±0.61	0.67±0.38
**A_{2A}AR/E169Q**	1.21±0.40	1.11±0.33	1.49±0.18	0.63±0.29

Table. S1 RMSD analysis of ZM241385, E(Q)169, H264, and T(A)256 in the A_{2A}AR, A_{2A}AR/E169Q, and A_{2A}AR/T256A structures.

RMSD were calculated with the initial structure of the MD simulation as the reference.

References

1. Y. Kofuku, T. Ueda, J. Okude, Y. Shiraishi, K. Kondo, M. Maeda, H. Tsujishita and I. Shimada, Efficacy of the β_2-adrenergic receptor is determined by conformational equilibrium in the transmembrane region, *Nat. Commun.*, 2012, **3**, 1045.
2. H. M. Weiss and R. Grisshammer, Purification and characterization of the human adenosine A(2a) receptor functionally expressed in *Escherichia coli*, *Eur. J. Biochem.*, 2002, **269**, 82-92.
3. W. Liu, E. Chun, A. A. Thompson, P. Chubukov, F. Xu, V. Katritch, G. W. Han, C. B. Roth, L. H. Heitman, A. P. IJzerman, V. Cherezov and R. C. Stevens, Structural basis for allosteric regulation of GPCRs by sodium ions, *Science*, 2012, **337**, 232-236.
4. C. C. G. ULC, Molecular Operating Environment (MOE), 2024.0601*Journal*, 2025.
5. M. J. Abraham, T. Murtola, R. Schulz, S. Páll, J. C. Smith, B. Hess and E. Lindahl, GROMACS: High performance molecular simulations through multi-level parallelism from laptops to supercomputers, *SoftwareX*, 2015, **1-2**, 19-25.
6. D. Case, H. Aktulga, K. Belfon, D. Cerutti, G. Cisneros, V. Cruzeiro, N. Forouzesh, T. Giese, A. Götz, H. Gohlke, S. Izadi, K. Kasavajhala, M. Kaymak, E. King, T. Kurtzman, T. Lee, P. Li, J. Liu, T. Luchko, ... and K. J. Merz, The AmberTools, *J. Chem. Inf. Model.*, 2023, **63**, 6183-6191.
7. J. Maier, C. Martinez, K. Kasavajhala, L. Wickstrom, K. Hauser and C. Simmerling, ff14SB: Improving the Accuracy of Protein Side Chain and Backbone Parameters from ff99SB, *J. Chem. Theory Comput.*, 2015, **11**, 3696-3713.
8. J. Wang, R. Wolf, J. Caldwell, P. Kollman and D. Case, Development and testing of a general amber force field, *J. Comput. Chem.*, 2004, **25**, 1157-1174.
9. M. J. Frisch, G. W. Trucks, H. B. Schlegel, G. E. Scuseria, M. A. Robb, J. R. Cheeseman, G. Scalmani, V. Barone, G. A. Petersson, H. Nakatsuji, X. Li, M. Caricato, A. V. Marenich, J. Bloino, B. G. Janesko, R. Gomperts, B. Mennucci, H. P. Hratchian, J. V. Ortiz, ... and D. J. Fox, Gaussian 16 Rev. C.01*Journal*, 2016.
10. K. Kitaura, E. Ikeo, T. Asada, T. Nakano and M. Uebayasi, Fragment molecular orbital method: an approximate computational method for large molecules, *Chem. Phys. Lett.*, 1999, **313**, 701-706.
11. Y. Mochizuki, S. Tanaka and K. Fukuzawa, *Recent advances of the fragment molecular orbital method : enhanced performance and applicability*, Elsevier, Singapore, 2021.
12. Y. Mochizuki, S. Koikegami, T. Nakano, S. Amari and K. Kitaura, Large scale MP2 calculations with fragment molecular orbital scheme, *Chem. Phys. Lett.*, 2004, **396**, 473-479.
13. Y. Mochizuki, T. Nakano, S. Koikegami, S. Tanimori, Y. Abe, U. Nagashima and K. Kitaura, A parallelized integral-direct second-order Moller-Plesset perturbation theory method with a fragment molecular orbital scheme, *Theor. Chem. Acc.*, 2004, **112**, 442-452.
14. D. Fedorov and K. Kitaura, Pair interaction energy decomposition analysis, *J. Comput. Chem.*, 2007, **28**, 222-237.
15. K. Fukuzawa and S. Tanaka, Fragment molecular orbital calculations for biomolecules, *Curr. Opin. Struct. Biol.*, 2022, **72**, 127-134.
16. D. Takaya, C. Watanabe, S. Nagase, K. Kamisaka, Y. Okiyama, H. Moriwaki, H. Yuki, T. Sato, N. Kurita, Y. Yagi, T. Takagi, N. Kawashita, K. Takaba, T. Ozawa, M.

Takimoto-Kamimura, S. Tanaka, K. Fukuzawa and T. Honma, FMODB: The World's First Database of Quantum Mechanical Calculations for Biomacromolecules Based on the Fragment Molecular Orbital Method, *J. Chem. Inf. Model.*, 2021, **61**, 777-794.
17. D. Guo, A. C. Pan, R. O. Dror, T. Mocking, R. Liu, L. H. Heitman, D. E. Shaw and A. P. IJzerman, Molecular basis of ligand dissociation from the adenosine A2A receptor, *Mol. Pharmacol.*, 2016, **89**, 485-491.
